# Hips Don’t Lie: Fong Disease

**DOI:** 10.5334/jbr-btr.1302

**Published:** 2017-04-26

**Authors:** Taj Mohammad Waziri, Annemieke Milants, Vikram rao Bollineni, Michel Maeseneer

**Affiliations:** 1 UZ Brussel, BE

**Keywords:** Nail patella syndrome, Illac horns, HOOD

A 61-year-old woman with history of chronic renal failure with secondary hyperparathyroidism and hypertensive retinopathy presented to the emergency department with the chief complaints of low back pain and mild bilateral knee pain. The pain was felt particularly while walking and was slowly progressive in nature. She also gave a history of repeated falling. Radiographic evaluation of the lumbar spine showed an acute compression fracture of TH 12 and an old fracture of both the right superior and right inferior pubic ramus.

An anteroposterior radiograph of the pelvis showed the presence of iliac horns on both sides (Figure 1A). A 3D reconstructed computed tomography (CT) of the pelvis displayed these horns as bony excrescences at the level of the iliac bones (Figure 1B). MRI showed that the gluteus medius muscle insertions was located at the level of iliac horns (Figure 1C). An axial radiographic view of the knee revealed both patellae to be hypoplastic. Based on the radiographic findings, the diagnosis of Fong disease was suggested.

**Figure 1A:**
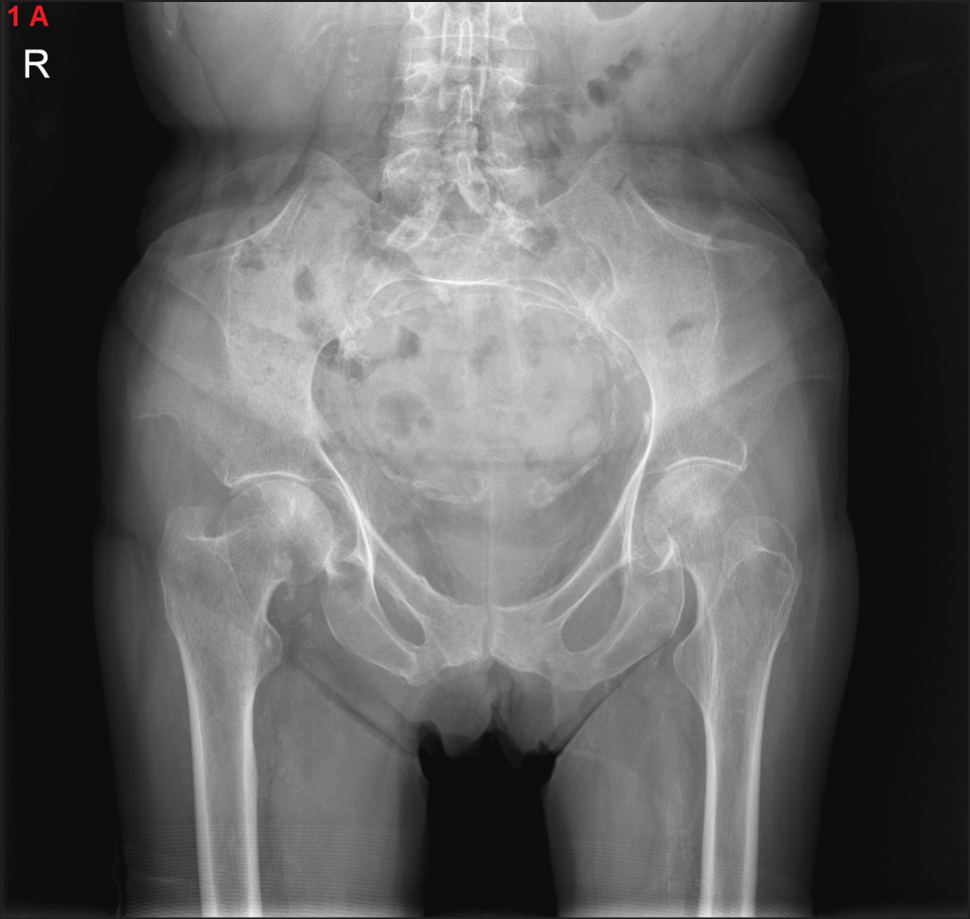
Anteroposterior radiograph of the pelvis shows the presence of bilateral triangular osseous outgrowth from the iliac bones.

**Figure 1B:**
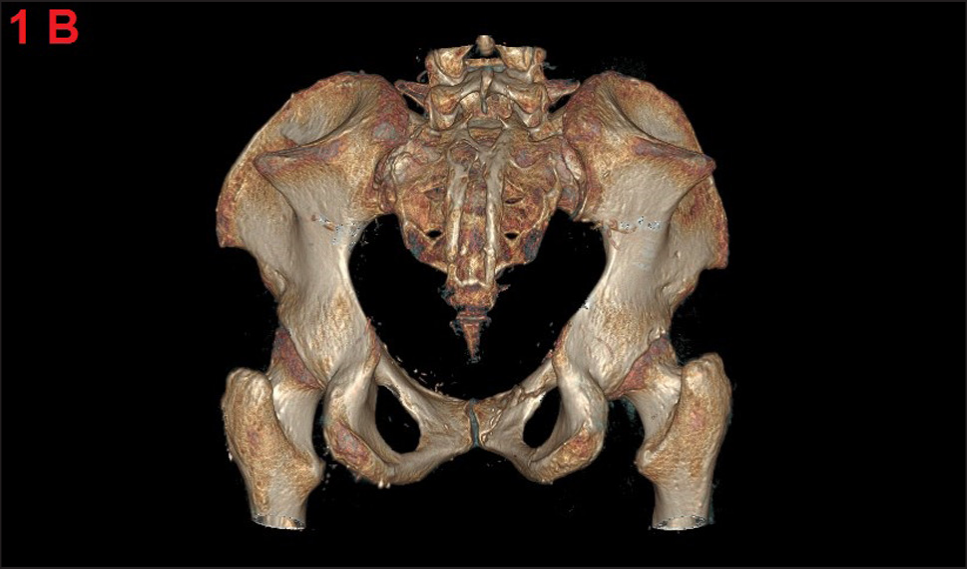
3D reconstruction image, posterior view, of the pelvic CT shows the bilateral iliac horns.

**Figure 1C:**
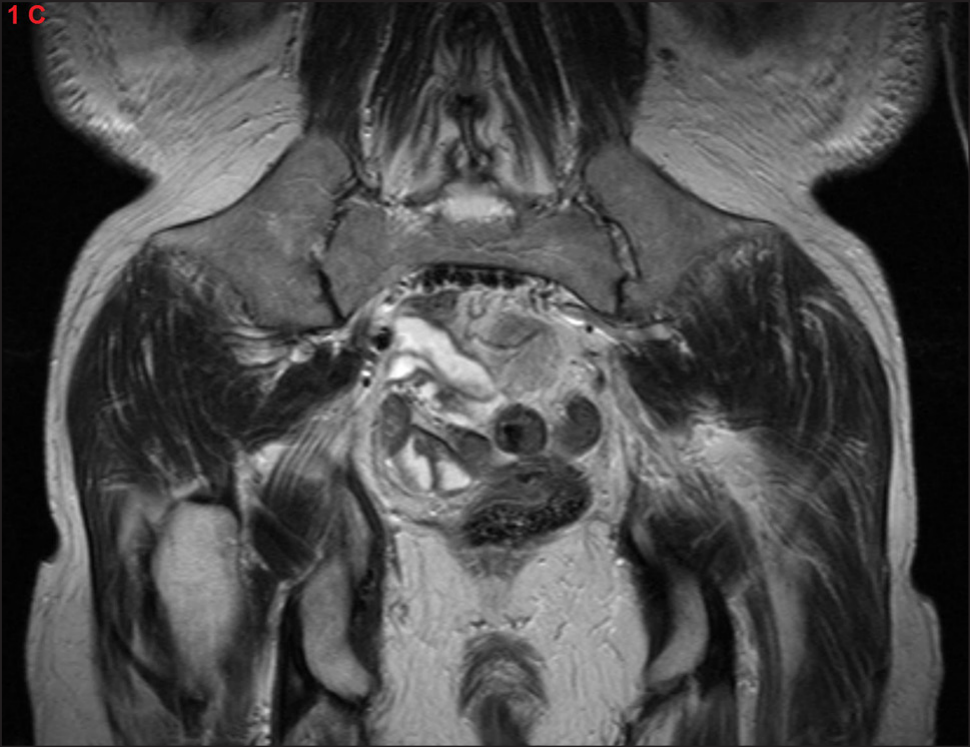
Coronal T2 image showing the gluteus medius muscle insertion at the level of the iliac horns.

## Comment

Fong disease, also known as hereditary onycho-osteodystrophy (HOOD) and commonly known as nail-patella syndrome (NPS), is a rare autosomal dominant hereditary disorder. Its incidence is approximately 1 in 50,000 new-borns. The underlying genetic defect has been localized at the LMX1B gene on chromosome 9 and the reported mean age of the diagnosis is 32 years [1]. Classically, patients present with a clinical tetrad: hypoplastic or absent patellae, finger nail dysplasia, dislocation of the radial head and the presence of iliac horns [1]. These posterior iliac horns are considered a pathognomonic sign of Fong disease and have been observed in 80% of cases. They are usually bilateral, cone-shaped osseous outgrowths that project dorsolaterally from the iliac bone. They act as the origin site of the gluteus medius muscles. Apart from physical manifestations, there is also a strong association with progressive nephropathy and open-angle glaucoma. Nephropathy is reported to be present in 30% to 60% of patients with progression to nephrotic syndrome in 20% and renal failure requiring dialysis or transplant in approximately 10%. Open-angle glaucoma occurs in 10%. One third of patients with Fong disease have problems with constipation or irritable bowel syndrome, and many exhibit sensory neuropathy.

There is no cure for NPS, and treatment is directed towards addressing the various orthopedic and non-orthopedic manifestations of the disorder. Patients with Fong disease seek medical attention for supposedly unrelated pathologies. In many cases, the radiologist may be the first to make the clinical diagnosis based on the radiographic findings. If the diagnosis of Fong disease is suggested, the presence of iliac horns can substantiate this presumption.
